# Are antipsychotics carcinogenic?: A review of the literature

**DOI:** 10.1192/j.eurpsy.2024.1337

**Published:** 2024-08-27

**Authors:** S. Walha, D. Mezri, K. Mahfoudh, S. Hamzaoui, A. Ouertani, U. Ouali, A. Aissa, R. Jomli

**Affiliations:** ”Psychiatry A”, Razi Hospital, Manouba, Tunisia

## Abstract

**Introduction:**

Antipsychotics are currently widely prescribed for various mental disorders. A presumption of a potential carcinogenic effect of antipsychotics was raised by certain studies. There are few data in the literature on this subject.

**Objectives:**

Study the relationship between the use of antipsychotics and the risk of cancer.

**Methods:**

A systematic literature review was carried out on PubMed looking for articles in English, published during the last decade (2013-2023), using the keywords “Antipsychotics” and “Cancer”. We included all articles studying the relationship between antipsychotics use and cancer risk.

**Results:**

Nine articles were included in our study, the majority of which focused on breast cancer. The results regarding breast cancer were discordant: although three studies did not show an association between the administration of antipsychotics and breast cancer, more recent studies have proven the opposite. Indeed, chronic exposure to antipsychotics, particularly those raising prolactinemia, was significantly associated with an accumulated risk of breast cancer, especially with positive estrogen receptors, whereas prolactin-sparing antipsychotics were not associated with it. Regarding hematologic malignancies, unlike other antipsychotics, long-term use of clozapine was associated with a high risk of malignancy, and had a greater effect on mortality from lymphoma and leukemia than to agranulocytosis. On the other hand, it has been proven that the use of atypical antipsychotics is associated with a reduced risk of lung cancer.

**Conclusions:**

Data from the literature regarding the carcinogenic potential of antipsychotics remain discordant and inconclusive. The most recent studies are worrying and highlight in particular an association between the use of antipsychotics and the increased risk of breast cancer. If these data are confirmed in future studies, this will undoubtedly impact the benefit-risk balance when making therapeutic decisions.

**Disclosure of Interest:**

None Declared

